# Ticks and Tick-Borne Diseases in Cuba, Half a Century of Scientific Research

**DOI:** 10.3390/pathogens9080616

**Published:** 2020-07-28

**Authors:** Dasiel Obregón Alvarez, Belkis Corona-González, Alina Rodríguez-Mallón, Islay Rodríguez Gonzalez, Pastor Alfonso, Angel A. Noda Ramos, Adrian A. Díaz-Sánchez, Maylin González Navarrete, Rafmary Rodríguez Fernández, Luis Méndez Mellor, Helen N. Catanese, Manuel Peláez, Yousmel Alemán Gainza, Roxana Marrero-Perera, Lisset Roblejo-Arias, Evelyn Lobo-Rivero, Claudia B. Silva, Adivaldo H. Fonseca, Eugenio Roque López, Alejandro Cabezas-Cruz

**Affiliations:** 1 School of Environmental Sciences, University of Guelph, Guelph, ON N1G 2W1, Canada; 2 Center for Nuclear Energy in Agriculture, University of Sao Paulo, Piracicaba, SP 13400-970, Brazil; 3 Direction of Animal Health, National Center for Animal and Plant Health, Carretera de Tapaste y Autopista Nacional, Apartado postal 10, San José de las Lajas, Mayabeque 32700, Cuba; bcorona@censa.edu.cu (B.C.-G.); pastor.alfonso@infomed.sld.cu (P.A.); rmarrero@censa.edu.cu (R.M.-P.); lroblejoarias90@gmail.com (L.R.-A.); elobo@censa.edu.cu (E.L.-R.); 4 Animal Biotechnology Department, Center for Genetic Engineering and Biotechnology, Avenue 31 between 158 and 190, P.O. Box 6162, Havana 10600, Cuba; alina.rodriguez@cigb.edu.cu; 5 Department of Mycology-Bacteriology, Institute of Tropical Medicine Pedro Kourí, Apartado Postal 601, Marianao 13, Havana 17100, Cuba; Islay@ipk.sld.cu (I.R.G.); angelnoda25@gmail.com (A.A.N.R.); 6 Department of Biology, University of Saskatchewan, 112 Science Place, Saskatoon, SK S7N 5E2, Canada; adiasanz88@gmail.com; 7 Department of Preventive Veterinary Medicine, Agrarian University of Havana, Carretera Tapaste y Autopista Nacional, Km 23½, Mayabeque 32700, Cuba; maylingo@unah.edu.cu (M.G.N.); roque@unah.edu.cu (E.R.L.); 8 National Laboratory of Parasitology, Ministry of Agriculture, Autopista San Antonio de los Baños, Km 1½, San Antonio de los Baños, Artemisa 38100, Cuba; rafmaryr123@gmail.com (R.R.F.); luis.mendez@lnp.art.minag.cu (L.M.M.); 9 School of Electrical Engineering and Computer Science, Washington State, University, Pullman, WA 99164, USA; helen.catanese@wsu.edu; 10 Direction of Animal Health, Ministry of Agriculture, Ave. Boyeros y Conill, Plaza, Havana 10600, Cuba; direpizootiologia@dsa.minag.gob.cu; 11 Faculty of Agricultural and Veterinary Sciences, Campus Jaboticabal, Via de Acesso Prof. Paulo Donato Castellane, Jaboticabal, São Paulo 14884-900, Brazil; yousmel@gmail.com; 12 Department of Animal Parasitology, Federal Rural University of Rio de Janeiro (UFRRJ), BR 465, Km 7, Seropedica, RJ 23890000, Brazil; claudia_ufrrj@yahoo.com.br; 13 Department of Epidemiology and Public Health, Federal Rural University of Rio de Janeiro (UFRRJ), BR 465, Km 7, Seropedica, RJ 23890000, Brazil; adivaldofonseca@yahoo.com; 14 UMR BIPAR, INRAE, ANSES, Ecole Nationale Vétérinaire d’Alfort, Université Paris-Est, 94700 Maisons-Alfort, France

**Keywords:** ticks, anaplasmosis, babesiosis, theileriosis, ehrlichiosis, Gavac^TM^

## Abstract

Ticks and the vast array of pathogens they transmit, including bacteria, viruses, protozoa, and helminths, constitute a growing burden for human and animal health worldwide. In Cuba, the major tropical island in the Caribbean, ticks are an important cause of vector-borne diseases affecting livestock production, pet animal health and, to a lesser extent, human health. The higher number of tick species in the country belong to the Argasidae family and, probably less known, is the presence of an autochthonous tick species in the island, *Ixodes capromydis*. Herein, we provide a comprehensive review of the ticks and tick-borne pathogens (TBPs) affecting animal and human health in Cuba. The review covers research results including ecophysiology of ticks, the epidemiology of TBPs, and the diagnostic tools used currently in the country for the surveillance of TBPs. We also introduce the programs implemented in the country for tick control and the biotechnology research applied to the development of anti-tick vaccines.

## 1. Introduction

The global growth of human population demands an increase in the production of food at the global scale [1]. Improving productivity is one way to increase livestock production on the existing suitable lands [2]. Ticks are hematophagous ectoparasites of vertebrates and represent an important impediment to livestock production in developing countries [3]. They also constitute a growing burden for human and pet animal health worldwide [4,5,6,7]. Besides causing direct damage associated with blood feeding and, in some cases, through the excretion of toxins within their saliva [8], the main relevance of ticks lies in the wide variety of pathogens they can transmit, including bacteria, viruses, protozoa, and helminths [9]. In the last 12 years, ticks have expanded to new areas and the incidence of tick-borne diseases (TBDs) has doubled [7]. The climatic change is driving the spread of ticks and transmitted pathogens to new regions [10,11,12]. A high proportion of TBDs are caused by co-infections, which further complicates the diagnostics and, in some cases, increases disease severity [7,13,14]. Without active tick control programs, many regions in the world, mainly in tropical areas, would be unable to sustain economically viable livestock production [15] and TBDs could become a more excruciating problem for human and animal health.

Cuba, the largest island in the Caribbean, is a developing country with scarce natural resources and incomes historically dependent on agriculture. As a tropical country, livestock production is greatly affected by ticks and TBDs. In Cuba, 34 tick species have been described [16,17] and within the Ixodidae family, four species are considered the most important from health and economic points of view: *Rhipicephalus microplus* (main host is cattle), *Rhipicephalus sanguineus* (dogs), *Dermacentor nitens* (horses) and *Amblyomma mixtum* (broad spectrum of mammalian host). Cuban cattle livestock inventory is approximately four million heads, mainly used for dairy purposes [18]. In natural conditions, more than 80% of ticks infesting cattle in the country are *R. microplus*, the main vector of *Babesia bovis*, *Babesia bigemina* and *Anaplasma marginale* to bovines [19,20] and buffaloes [21]. Due to the climatic conditions of Cuba, with high temperature and humidity, the free-living stages of *R. microplus* survive throughout the year and can complete up to four generations in a single year [22]. Larval survival rates are especially high on pasture during the rainy season, from May to November [23]. In these circumstances, tick eradication is impracticable.

Likewise, ticks and TBDs pose a threat to other animals in Cuba, especially horses, small ruminants, and pets [24,25]. No significant incidence of TBDs has been reported in humans in the country. However, this is an active area of research in Cuba due to the importance of this threat to public health worldwide and the potential risk that this problem may rise in the future [26,27,28]. Several institutions have worked during the last 50 years in the study of ecoepidemiology of TBDs, as well as in their prevention and control in the country, achieving meritorious results in many research areas. Remarkably, Cuba produces the only commercially available vaccine used for tick control, Gavac^TM^ (Heber Biotec, Havana, Cuba). During this period, there was a close inter-institutional and inter-sectoral relationship, which allowed for a multidisciplinary approach to the study of ticks and TBDs in the country. A vast catalog of scientific publications has been produced on this topic. However, many of them are only available in Spanish language, printed journals, and are distributed only in Cuba and Latin America, which impairs the wide access of the scientific community to this valuable literature. In this review, we covered major areas of tick and TBD research in Cuba, mainly in the period spanning from 1970 to 2020.

## 2. Tick Species in Cuba

Ticks (Acari: Ixodida) are grouped into three families, Argasidae, Ixodidae and Nuttalliellidae, 17 genera, and more than 930 species and species complex [29,30]. Recently, an extinct tick family (Deinocrotonidae) that fed on dinosaurs was described [31]. Tick classification is based on the description of morphological and genetic traits [32].

The study of tick diversity in Cuban can be separated in three periods: (i) 1930–1956, with studies led by Dr. Ildelfonso Perez-Vigueras [33], who made the first catalog of tick species of Cuba [34,35,36], (ii) 1964–1968, these studies were updated and extended in the framework of cooperation between Cuban and Czechoslovak institutions [37,38], and (iii) 1973–1995, characterized by the continuous studies performed by de la Cruz et al. [39,40,41,42,43,44,45,46,47]. Afterwards, studies on this topic have been scarce. The tick species reported in Cuba include 25 and 9 members of the families Argasidae (Table 1) and Ixodidae (Table 2), respectively. With few exceptions, tick species classification in Cuba has been based on morphological traits. The Cuban provinces with more tick species diversity are Pinar del Río (10), Mayabeque (9), Artemisa (8), Isla de la Juventud (8) and Camagüey (7). Hosts carrying the majority of tick species are bats (18), other mammals (e.g., domestic dogs, equines, bovines) (6), and reptiles (5).

The diversity of Cuban ticks illustrates similarities with the tick fauna of the neighboring islands (i.e., Greater Antilles), which are considered biodiversity hotspots, with an abundance of endemic species [16]. *I. capromydis* is a Cuban endemic tick species with noteworthy ecological importance reported only in the Isla de La Juventud [48], in spite of the fact that the host (*Capromys pilorides*, Rodentia: Capromyidae) is widespread in the country [39]. To summarize, studies on tick systematics in Cuba contributed to the tick research community through the description of 34 tick species, one of them endemic, and their ecology. However, molecular studies are needed to achieve more precise taxonomical descriptions and to further characterize the genetic diversity of ticks in the Island.

## 3. Ecophysiology of Ticks in Cuba 

In the early 1970s, several investigations were carried out on the ecology and adaptation of tick species to the climatic conditions of Cuba [22,52]. These studies were systematized by Dr. Rafael de la Vega Ruibal (1934–2012) [53] and his collaborators. The main focus of de la Vega’s research was the effect of thermal constants on the development of *R. microplus* ticks [54,55,56,57,58,59,60]. The results showed that the most favorable conditions for the development and reproduction of *R. microplus* were 30 °C of temperature and 100% of relative humidity, while the combination of higher temperatures (i.e., 32 and 34 °C) and lower relative humidity (i.e., 70% and 75%) reduced the performance of this tick species. This explained how the climatic conditions of Cuba, with balanced temperature and humidity, supports the presence of *R. microplus* throughout the year [61,62]. In other studies, de la Vega and Díaz [58,59] estimated thermal constants for the optimal hatchability of *R. microplus* egg under simulated natural conditions, and its relation to the period of time between detaching of engorged females and egg hatching. Following these results, rotational grazing was organized to shorten paddock occupation times during the rainy season (May to November), in order to avoid the presence of cattle in paddocks during the optimal period for egg hatching. The effect of thermal constants on the non-parasitic stage of *D. nitens* was also studied [63,64]. The most favorable climatic conditions for *D. nitens* in Cuba were temperatures between 26 and 30 °C and 100% of relative humidity [63,65,66]. The studies by de la Vega et al. [32] showed that the non-parasitic stage of *R. microplus* was better adapted to the warm-humid conditions of Cuba than that of *D. nitens*.

The host species (i.e., cattle or horse) was found to influence the weight and reproductive performance of fully engorged *D. nitens* female ticks [67]. Particularly, the weight of engorged females that fed on equines was significantly higher than those that fed on cattle [67]. Consequently, the number of eggs produced by ticks that fed on equines was higher than those fed on bovines. The authors also found that feeding on cattle or horse did not affect the duration of the non-parasitic life stage of *D. nitens* [67].

The tick infestation of cattle with different genotypes (i.e., F1 Holstein x Zebu) was compared with that of water buffalo raised in Matanzas province, Cuba [68]. *A. mixtum* was found to be the main tick species infesting cattle, followed by *R. microplus*. Only *A. mixtum* was found on buffaloes and the tick infestation burden on this species was lower compared with cattle. However, in Mayabeque province, Obregón et al. [69] found that 100% of ticks collected from buffaloes were *R. microplus*. These results support the effect of topological and climatic conditions on the distribution of tick species, especially for *A. mixtum*, which is only found in certain regions of the country [17]. Interestingly, the infestation by *R. microplus* tick is scarce in adult buffaloes, even when these animals are reared close to bovines. However, the prevalence of ticks in infested buffalo calves was similar to that of adult and calf cattle [69,70]. It was also observed that the tick burden in buffalo calves was higher when raised in immediate proximity to cattle herds, although the larval survival rate was higher in cattle calves than in buffalo calves [70].

The first comprehensive characterization of *R. sanguineus* in Cuba was in 2015, when the sensitivity to different acaricides and the molecular and morphological features of a field strain of this tick species were characterized [71]. The Cuban strain was closely related to *R. sanguineus* sensu lato (s.l.) belonging to the tropical lineage and was resistant to amitraz. The life cycle was also characterized, and it lasted 85 days under laboratory conditions [71]. Subsequently, the Cuban strain of *R. sanguineus* s.l. was confirmed to be genetically similar to other "tropical strains" from Brazil, Thailand, Mexico, Colombia, South Africa, and Mozambique, and all formed a clade separated from the "temperate strains", such as those found in Argentina, Spain, and the United States. Additionally, the analysis of biological parameters showed that engorged females from the temperate strains were heavier than engorged females from the tropical strains. Furthermore, among the tropical strains, the *R. sanguineus* s.l. engorged females from Cuba were 35% and 48% lighter than those from Brazil and Thailand, respectively [72].

## 4. TBDs in Humans

TBDs are not considered a major problem for human health in Cuba, probably due to limited information about their incidence in the country. In contrast to other zoonotic diseases, tick-borne zoonoses are not recognized, nor clinically suspected by the Cuban physicians. However, historical reports of TBDs in companion and farm animals in Cuba, and the zoonotic risk of TBPs, justify their study under the ‘One Health’ strategy.

A survey about Lyme disease knowledge applied to Cuban physicians; mostly dermatologists, general clinicians, and epidemiologists; from the different health attention levels revealed that 70% of the doctors knew about Lyme borreliosis, but only 46% recognized at least one of its clinical manifestations [27]. Lyme disease is the only TBD for which a laboratory diagnosis has been established in Cuba. Since the 1980s, some physicians and researchers suspected the presence of Lyme disease in Cuba [73]. Particularly, several individuals from a rural village in Pinar del Río, with reports of high levels of *A. mixtum* infestation, especially in children, were hospitalized with clinical suspicion of Lyme borreliosis, but, unfortunately, the laboratory diagnosis was not available at that the time to test for *Borrelia* infection [73]. 

The most probable case of *Borrelia* infection in Cuba was a biologist, specializing in ticks, who had never traveled to another country and had suffered ixodid bites in numerous field expeditions. During early 1984, he had severe neurological disorders diagnosed as a myeloradiculitis or Guillain Barré syndrome; but it was not until 1987, when he traveled to Czech Republic, that neuroborreliosis was retrospectively diagnosed using an immunofluorescence assay (IFA) for *Borrelia burgdorferi* s.l. [74]. During 1998–2002, another two cases out of 14 patients, from the same village of Sierra del Rosario in Pinar del Río province mentioned above, were confirmed as having IgM specific antibodies to *B. burgdorferi* measured by enzyme-linked immunosorbent assay (ELISA) and Western blot [74]. One of them was an adolescent who was reported with clinical and epidemiological suspicion of Lyme disease when he was a child [73,74]. Since 2003, IgM and IgG specific to *B. burgdorferi* were detected in another 16 patients with a history of tick bites and clinical evidence of Lyme borreliosis in different regions of Cuba [27,75]. The number of samples (mainly serum and cerebrospinal fluid) received by the National Reference Laboratory to perform Lyme disease diagnosis has increased in recent years [27]. Specific anti-*Borrelia* IgG were estimated between 0.6–7.2% of the population living in the village of Sierra del Rosario, who are frequently exposed to tick bites [76]. However, no anti-*Borrelia* antibodies were detected in a blood donor cohort from an urban region with low tick infestation, suggesting that the population of Sierra del Rosario has been exposed to *Borrelia* spp. and that the detected antibodies are not part of a non-specific background reactivity, as suggested by other authors [28].

Exposure to other TBPs were explored in the same population of Sierra del Rosario and antibodies to *Anaplasma phagocytophilum*, *Erhlichia chaffensis* and *Babesia microti* were detected in 7.2%, 3.6%, and 11.5% of studied serum samples, respectively [77]. Interestingly, an outbreak of bovine babesiosis was associated with the antibody detection against *Babesia* spp. in farmers of Ciego de Avila, a province in central Cuba [78]. Antibodies against *B. bovis* and *B. bigemina* antigens were detected by IFA in 7% and 3.9%, respectively, of blood donors of the same locality [78]. The detection of zoonotic pathogens in ticks collected on bovines, equines and canines has been an indirect strategy to assess the risk of human infections in the country. The results highlight the importance of domestic animals in the epidemiological cycles of TBPs. *Anaplasma* spp./*Ehrlichia* spp. and *Babesia* spp. have been reported in hard ticks collected on pet animals in close contact with humans, although the identification of the tick species was not possible [26,75].

Diseases caused by rickettsial agents have not been reported yet in Cuba. However, *Rickettsia amblyommi*, a pathogen with controversial pathogenic risk for humans, has been detected in *A. mixtum* ticks collected on horses, dogs [79], and a human [80]. These findings suggest that individuals with cutaneous rash, fever and a history of tick bites should be carefully considered as TBD patients by physicians during a clinical examination. Q fever, caused by *Coxiella burnetii*, has not been reported in Cubans, but it is an interesting subject of research because this bacterium was detected in *A. mixtum* ticks collected on horses [79]. The molecular findings of zoonotic pathogens in *A. mixtum*, a highly anthropophilic tick, justify TBPs surveillance in Cuban populations at risk.

## 5. Epidemiology of Cattle Tick Fever in Cuba

**Figure 1:**
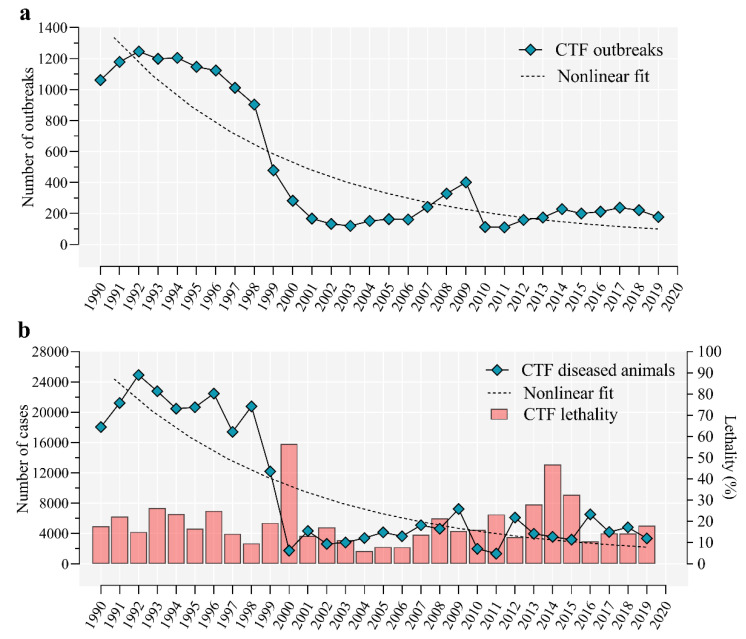
Time trend graph of cattle tick fever (CTF) in Cuba from 1990 to 2019. The figure displays (**a**) the number of outbreaks and (**b**) number of CTF cases and lethality by year. Dashed lines show the goodness-of-fit of the non-linear regression model.

Cattle tick fever (CTF), a complex of diseases including babesiosis and anaplasmosis caused by *B. bovis*, *B. bigemina* and *A. marginale*, respectively is the most important TBD affecting cattle and buffaloes in Cuba [81,82]. Notably, *B. bovis*, *B. bigemina* and *A. marginale*, transmitted by *R. microplus*, are frequently found co-infecting cattle in endemic areas from South and Central America and the Caribbean region [19,81,83,84,85]. The CTF is endemic in Cuba [86]. A long-term and intensive study of TBDs in Cuba from 1968 to 1987 revealed that 1631 outbreaks (total: 1702, 95.83%) were due to anaplasmosis or babesiosis [87]. However, in the early ‘90s, an upsurge in CTF was observed in the country, with significant economic losses and reduction in the cattle population. According to statistics from the National System for Information and Epizootiological Surveillance (SIVE) from January 1990 to December 2019 [88], the dynamics of CTF outbreaks (Figure 1a), and the number of cases per year (Figure 1b), revealed a clear dichotomous pattern. In the ‘90s, the number of CTF outbreaks averaged 1055 ± 227 with an incidence of 20,086 ± 3572 cases per year, while a sharp fivefold decrease was observed in 2000 which remains until today, underlying the endemic stability of CTF during the last 20 years in Cuba. In addition, the percentage of lethality averaged 18.68%, (6% to 46.81%) in the last 30 years with highest levels in 2000 and 2014 (Figure 1b). However, considering the incidence of CTF, the annual average of dead animals due to CTF from the 90s was 5.7 times higher than in the last 20 years.

The epidemy of CTF in the ‘90s overlaps with a period of a severe economic crisis in Cuba that limited resources for livestock farming [89]. The situation was particularly challenging because when the crisis began in 1990, 70% of the cattle population was Tropical Holstein (31/32 Holsteins × 1/32 Zebu) [90], which are highly susceptible to tick infestation and pathogens causing CTF [91,92,93]. The remarkable and sustained reduction in the number of CTF cases from the year 2000 onwards could be explained by several reasons including (i) the substitution of the highly productive Holstein breed by the more robust ‘Siboney de Cuba’ (5/8 Holstein × 3/8 Zebu) [90], (ii) changes in cattle breeding practices, specifically the elimination of the artificial rearing of calves, which restricted their contact with the environment, therefore hindering the development of a long-term protective immunity [94,95,96], and (iii) the implementation of the Cuban National Program for Integrated Tick Control (CNPITC) in 1996 (discussed later in this review).

## 6. Ticks and TBPs Infection in Water Buffalo

Water buffalo are a relatively new livestock variety in Cuba, with a population of around 2,000 animals introduced to the country in 1989 [97]. Currently, there are over 60,000 animals spread all over the country, usually in grazing areas in close proximity to cattle herds [98]. The buffaloes are also infested by *R. microplus* [69] and *A. mixtum* [68] in the Cuban grassland. The buffalo calves are most affected by ticks, registering parasitic loads similar to cattle [69,70]. Furthermore, although the survival rate of *R. microplus* larvae on buffaloes is lower than on cattle, engorged females fed on buffaloes have a reproductive performance similar to ticks fed on cattle [70].

In Cuba, no clinical cases of CTF have been detected in buffaloes. However, epidemiological studies using molecular (i.e., Polymerase chain reaction PCR and nested (n) PCR) and serological (i.e., indirect (i) ELISA) diagnostic tests found a prevalence greater than 50% for *B. bovis*, *B. bigemina* and *A. marginale* in buffaloes of the western region of Cuba [21,99]. Prevalence and infection levels found in water buffaloes suggest that these animals are potential reservoirs of CTF pathogens in the ecoepidemiological conditions of Cuba, especially in areas where cattle and buffaloes coexist [100,101,102]. Remarkably, *A. marginale* strains infecting buffalo were genetically related to those infecting cattle in the same region [100], suggesting circulation of this pathogen between buffalo and cattle. Furthermore, the transovarial transmission of *B. bovis* and *B. bigemina* in *R. microplus* engorged female ticks fed on buffalo suggests that this host species can support the life cycle of some TBPs in Cuba [70]. Nevertheless, the reservoir capacity of buffaloes for CTF pathogens, and the role of these animals in the epidemiological process of CTF disease across the country, should be further evaluated.

## 7. Ticks and TBPs Infection in Horses

Equine piroplasmosis is an economically important tick-borne protozoal disease of horses, mules, donkeys, and zebras that is characterized by acute hemolytic anemia [103]. The etiologic agents are two protozoan parasites of the phylum Apicomplexa, *Theileria equi,* and *Babesia caballi* that are transmitted primarily by ixodid ticks of the genera *Rhipicephalus*, *Dermacentor*, *Haemaphysalis*, and *Hyalomma* [104]. Equine piroplasmosis is a notifiable disease by the World Organization for Animal Health (OIE) with a worldwide distribution that is endemic in tropical, subtropical, and some temperate regions, where competent tick vectors are present [105]. This disease represents a serious problem for the horse industry due to the cost of treatments, abortions, loss of performance, death, and restrictions to the international movement of horses for trade or international equestrian events [106].

The occurrence of equine piroplasmosis within the Caribbean region is still controversial, although the presence of both etiologic agents *T. equi* and *B. caballi* have been reported on most of the Caribbean islands, including Cuba [107,108]. The first report of equine piroplasmosis in Cuba was conducted by Salabarria, et al. [109], which detected the coinfection of both *T. equi* and *B. caballi* in clinically healthy zebras imported into Cuba from South Africa. Further studies in the early ‘80s confirmed the occurrence of *T. equi* and *B. caballi* infections in Cuban equine herds using microscopy examination and the complement fixation test (CFT) [107,110]. Based on these findings, a national program for surveillance and control of equine piroplasmosis was implemented in Cuba, including a systematic (yearly) serological survey over the equine population across the country.

The first molecular evidence of *T. equi* and *B. caballi* infections in Cuban horses including nPCR diagnosis, DNA sequencing and strain phylogenetic analysis was reported in 2018 [24]. In that study, the overall prevalence of piroplasm infection was 78%, with 73% for *T. equi*, 25% for *B. caballi*, and 20% of coinfections, suggesting that these pathogens are endemic in Cuba. The phylogenetic analyses based on the *T. equi* 18S rRNA gene revealed the presence of genotypes A and C in the Cuban horses, which is consistent with reports from the Caribbean, as well as North and South America [111,112,113]. Further studies are needed to identify the distribution of competent tick vector, the risk factors associated to infection spread, and to explore the genetic diversity of *T. equi* and *B. caballi* in the equine population of Cuba.

## 8. Ticks and TBPs Infection in Dogs

Dogs are a potential reservoir of zoonotic TBPs with a major impact on animal and public health. The first published studies on hemoparasitic infection of dogs in Cuba were carried out by Pérez et al. [114], who described a case of canine ehrlichiosis, caused by *Ehrlichia canis*, based on clinical and pathological findings. Years later, León et al. [115] studied 155 dogs with a history of tick infestation and observed rickettsia-like structures in blood smears from 13 of them. In addition, 82.5% of animals were seropositive for *E. canis* [115]. This study also described clinical signs of the disease and explored the risk factors associated with the infection of this pathogen. 

More recently, an epidemiological study using molecular (i.e., PCR) and serological (i.e., iELISA) tests found 50% of *E. canis* positive samples and 78.6% of *E. canis* seroprevalence, respectively, among domestic dogs (*n* = 162) from four municipalities in the western region of Cuba [25]. Additionally, a study by Navarrete et al. [24] revealed an increased risk of *Ehrlichia* infection in some localities with a history of tick infestation. The presence of *A. platys*, the agent of canine cyclic thrombocytopenia, was also confirmed by PCR diagnosis in 16% of tested dogs (*n* = 100) [116], and the observation of *A. platys*-like morulae in dog platelets by using blood smears [116].

Tick infestation on dogs was assessed in the western region of Cuba revealing that 40% of dogs (*n* = 378) were infested by ticks that were morphologically characterized as *R. sanguineus* s.l. [117]. In addition, 49 pools according life stages and municipalities of collected *R. sanguineus* ticks were screened for other TB pathogens through PCR assays. The pools were positive for *E. canis* (8.9%), *A. platys* (10.2%) and *Babesia canis vogeli* (2.04%) [117]. Subsequently, an epidemiological study conducted in the same municipalities detected a high prevalence of *E. canis* in dogs, which provided strong evidence that *R. sanguineus* is the vector of *E. canis* in Cuba [118]. These results constituted the first molecular evidence of *E. canis*, *A. platys* and *B. canis vogeli* presence in ticks infesting dogs in Cuba. Hence, further studies should be conducted to analyze the vectorial capacity of *R. sanguineus* for these pathogens, as well as the epidemiological factors driving TBDs in dogs in Cuba.

## 9. TBPs Infection in Sheep and Goats

Over the past decade, much of the focus of TBDs research of livestock in Cuba has been directed toward bovine pathogens. The small ruminants have received relatively limited attention despite sheep and goats being an important source of food and involve state production and private farmers across the country [119,120]. The high genetic value of the sheep herds, mainly composed of the Pelibuey breed (also known as ‘Cubano Rojo’) [121,122], could be associated with high incidence of infectious diseases, including TBDs.

Sheep and goats are affected by several TBDs worldwide, including anaplasmosis and babesiosis, mainly caused by *Anaplasma ovis* and *Babesia ovis.* However, these TBPs are often underestimated in these livestock because the diseases they cause are mainly subclinical, and there is a natural resistance acquired by autochthonous species in endemic countries [123]. In the Caribbean region, several TBPs have been reported infecting sheep and goats, such as *Rickettsia africae*, *B. caballi*, *B. canis rossi*, *B. canis vogeli, B. gibsoni, B. vulpes*, and *Theileria* spp. [85]. Especially, the very pathogenic dermatophilosis, caused by *Dermatophilus congolensis*, and the caprine heartwater, caused by *Ehrlichia ruminantium*, affect the small ruminants in the region [124]. Both TBPs are transmitted by *Amblyomma variegatum* ticks, which, despite not being present in Cuba (Table 2), after introduction in Guadeloupe 170 years ago, have spread in the Lesser Antilles, and pose a risk for both Cuba and North America [125].

Research conducted in Cuba [126], has described the infection of *Mycoplasma ovis* (formerly *Eperythrozoon ovis*) in sheep and goats. This hemotropic bacterium is transmitted by blood-feeding arthropods [127], with published research supporting tick transmission [128]. Rodriguez et al. [129] using blood smears from sheep detected infections by *A. ovis*, *B. ovis*, *M. ovis*, and *B. motasi*, as well as co-infections by *A. ovis*/*B. ovis*, *A. ovis*/*E. ovis*, and *A. ovis*/*B. motasi*. The parasites were detected in blood collected from the heart of sheep that had died or had been slaughtered. However, no studies have been conducted using sensitive and specific molecular methods to characterize the infection by these pathogens in sheep and goats in Cuba. Consequently, the prevalence and geographical distribution of TBPs in small ruminants across the country is a lacking information on the SIVE. These issues should be addressed, considering the strategic importance of the small ruminant production for the socio-economic development of Cuba.

## 10. Development of Diagnostic Tools for TBPs in Cuba 

Due to their economic impact, seven major animal TBPs are actively monitored in the Caribbean: *A. marginale*, *E. ruminantium*, *B. bovis*, *B. bigemina*, *B. caballi*, *T. equi* [84], and *B. burgdorferi* [74,76]. It is considered that the successful management of TBPs in livestock and public health depends on adequate knowledge of prevalence and the risk factors associated with pathogens spread [19,20,27,130]. Concerning livestock, a surveillance system for TBDs has been in place in Cuba since 1987, especially focused on CTF [131]. For that reason, a priority in Cuba has been the development of diagnostic tools for detection and monitoring of the major TBPs affecting livestock production and public health. This activity has been a prioritized research line at the National Center for Animal and Plant Health (CENSA), and the Institute of Tropical Medicine Pedro Kourí. 

Since the seventies of the last century, Giemsa-stained blood smear has been used as diagnosis method of CTF pathogens [132,133], and is the main diagnostic method used in the laboratory network of veterinary services in Cuba. However, while Giemsa staining is useful to confirm infection in animals with clinical disease, this method does not allow the identification of carrier animals due to low levels of infected erythrocytes/mL blood that are undetectable by blood smear, posing a challenge to the direct diagnosis that currently requires PCR assays [99,134]. Several diagnostic tests have been developed and used during the last 50 years in Cuba (Table 3), which have evolved according to the technological development of the country and the world.

Early studies in Cuba used morphological diagnosis, card agglutination test (CAT), and CFT, enabling the detection of several TBPs. Serological methods were introduced, increasing the scope of epidemiological studies, which reported seroprevalence of TBPs in cattle host [86,135,136,137]. The ELISA and IFA tests have been used successfully in the survey of specific antibodies to *B. bovis,* revealing consistent results from both serological tests [135]. An IFA, standardized in Cuba, was probably the most widely used test for the detection of antibodies to *Babesia* spp. [136]. However, reading the IFA slides using the microscope is subjective and vulnerable to experimenter bias [135]. The immunoperoxidase test was also used in the Cuban SIVE program, with results similar to those obtained by ELISA [137].

More recently, DNA-based diagnostic tests have been developed to screen CTF pathogens in Cuba. Corona et al. [139] developed a PCR assay for the diagnosis of *A. marginale* and found a high number of positive bovines without clinical symptoms of anaplasmosis. The multiplex qPCR described by Díaz-Sánchez et al. [14] proved to be a rapid, specific and cost-effective means for the simultaneous detection of *A. marginale* and *T. annulata*, confirming a high prevalence of *A. marginale* infection of carrier cattle in Cuba. On the other hand, a SYBR Green-based real-time PCR system developed for the detection and quantification of *B. bovis* and *B. bigemina* [101] detected low parasitemia levels in carrier buffaloes, and *R. microplus* tick larvae [70]. Likewise, a TaqMan-based real-time PCR assay enabled exploration of the reservoir competence of water buffalo for *A. marginale* in endemic areas of Cuba [100]. The combination of nPCR and iELISA assays was a useful approach used to determine the molecular and serological prevalence of *A. marginale*, *B. bovis*, and *B. bigemina* in water buffalo herds of western Cuba [21].

## 11. Genetic Variability of *A. marginale* and Other TBPs in Cuba

*A. marginale* causes bovine anaplasmosis and is a widespread TBP associated with significant economic losses in the cattle industry. The genetic diversity of *A. marginale* worldwide is likely explained by vector ecology and the international livestock trade [142]. The major surface protein 1 alpha (MSP1a) is a 70–100 kDa protein encoded by a single-copy gene *msp1a.* This gene is conserved during the bacterial multiplication in cattle and ticks; thus, *msp1a* sequence is recognized as a stable genetic marker for geographic strain identity [143]. This gene allows traceability of pathogen transmission, and provides insights into the evolution of vector-host-pathogen interactions [144,145]. Identification of *A. marginale* strains is possible due to a variable number of tandem 23–31 amino acid repeats (TRs) located in the N-terminal region of MSP1a, which are encoded by short sequence DNA repeats (SSRs) (84–87 bp) that typically occur two or more times in the *msp1a* sequence. Presumably, SSRs have a low mutation rate and remained the same in a given genotype across time [146]. To date, over 300 TRs have been reported [147].

**Figure 2:**
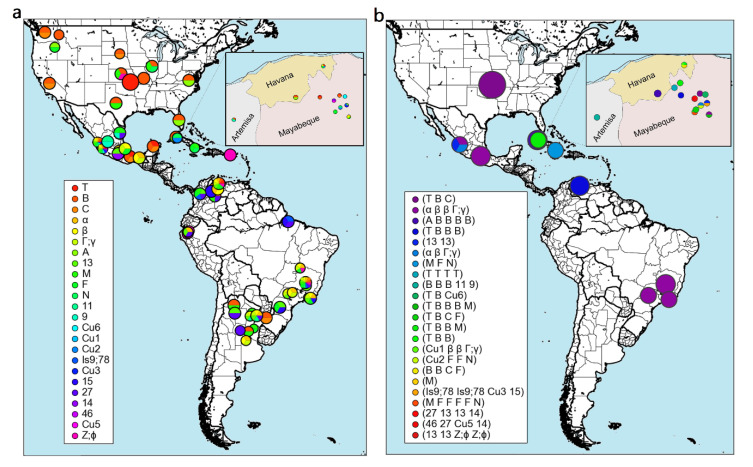
Geographic distribution of *A. marginale* strains found in Cuba, and their distribution in the Americas. The 37 major surface protein 1 alpha (MSP1a) sequences available in the GenBank from Cuba were included in the analysis, these from the Cuban provinces Artemisa, Mayabeque, Havana, and one isolate from Granma [14,100,148]. Panels represent the location of (**a**) tandem repeats (TRs) and (**b**) strains. The size of a pie chart indicates the scope of the region it denotes. The figures were automatically generated using RepeatAnalyzer software [147].

In this review, a metanalysis of the genetic diversity of *A. marginale* in Cuba and other regions of South and North America, based on *msp1a*, was performed. The study included 37 MSP1a isolates from Cuba. The analysis was made using the RepeatAnalyzer software [147]. The distribution of MSP1a TR and *A. marginale* strains in the Americas shows that most of the 24 TR found in *A. marginale* strains of Cuba were also identified in other regions of South and North America. Five of the TR (Cu1, Cu2, Cu3, Cu5, and Cu6) were identified only in the Cuban strains (Figure 2a). The molecular analysis of MSP1a allowed identification of 23 different strains of *A. marginale* in Cuba, some found only in Cuba, but most of them found also in Brazil, Venezuela, Mexico, and the United States (Figure 2b).

This suggests multiple introductions of *A. marginale* strains in Cuba through the importation of carrier cattle. The presence of *A. marginale* strains in Cuba not found in America can be explained by the origin of other cattle imports in Cuba [100], mostly from Europe and Africa. The discovery of unique TR, and the high diversity of strains in the small geographical area of western Cuba may suggest an accelerated mutation rate, which is consistent with the selective pressure exerted on the pathogen by the vector and immunological system of the host [143]. Several tick transmission cycles throughout the year, as occurs in Cuba, have been associated with high genetic diversity of *A. marginale* [142,144,145,149].

**Figure 3:**
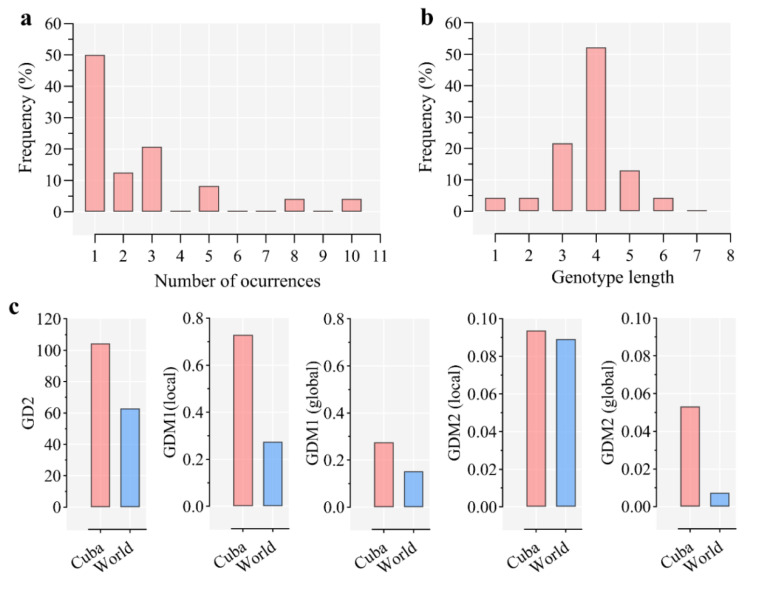
Genetic diversity analysis of *A. marginale* strains in western Cuba. (**a**) TRs frequency. (**b**) Genotype length distributions. **(c**) Genetic diversity (GD) metrics compared between strains from Cuba and world; GD2, represents the ratio between unique SSRs and the number of strains in a region; GDM1 also considers the number of unique SSRs in a region but is independent of the length of the genotype; and GDM2 measures how uniformly the SSRs occurrences in a region are distributed. GDM1 and GDM2 are presented in both local and global variants, in which the metrics are calculated as an average of the values for each genotype or over the entire region, respectively [147].

The TRs identified in *A. marginale* present in Cuba averaged 28.3 ± 1.72 amino acids, and 50% of the TRs were found only once in the analyzed strains. Five of the TRs were novel compared with other regions of the world, whereas the TRs T and B were the most common, found in nine and ten strains respectively (Figure 3a). Most *A. marginale* strains in Cuba (52%) had four TR (Figure 3b). Genetic diversity analysis was carried out comparing the *A. marginale* diversity scores from Cuba and the world (Figure 3c), as previously described [147]. The results showed that the number of unique TR in the region is almost double the world average, furthermore, the uniformity of distribution of TR among the strains is also higher in western Cuba (Figure 3c). Therefore, the high genetic diversity of the *msp1a* gene confirms the frequent circulation and evolution of *A. marginale* in western Cuba, and quite possibly throughout the country. This should be taken into account in future livestock development strategies [150], especially when it comes to breeding susceptible *Bos taurus* breeds [93,151,152].

Concerning other pathogens, molecular characterization of 21 isolates of *A. platys* detected in dogs in Cuba was performed based on *gltA* and 16S rRNA genes [116]. Likewise, eight isolates of *E. canis* detected in ticks fed on dogs were assessed using the16S rRNA sequences [117]. The analyzed sequences showed 99–100% identity with sequences of *A. platys* and *E. canis* available in GenBank database and reported in different regions of the world. Three different *A. platys* genotypes were identified in the dog blood samples. Some of the isolates of *A. platys* had a common ancestry with an African isolate, while others were related to isolates worldwide, suggestive of possible multiple introductions of this pathogen in Cuba with the influx of people, animals, and products [116]. Concerning *Babesia* sp. isolated in dogs, sequence analysis of the 18S rRNA gene had 100% identity when compared to sequences of *B. canis vogeli* deposited in the Genbank [117]. These results demonstrate the need to establish a control and prevention program to deal with tick-borne hemoparasitic diseases that affect dogs in Cuba.

## 12. Applications of Biotechnology in the Control of ticks in Cuba 

The idea that vaccines are potentially safer, cheaper and more efficacious as prophylactic than acaricides is based mainly on our experience with anti-microbial vaccines [153]. However, due to the biochemical complexity of parasites, such as ticks, the presence of multiple stages in their life cycle, the high number of different tick species, their capacity to parasitize every class of terrestrial vertebrates [154], and their contact with the host immune system only during feeding, the tick control using antigen immunizations is a challenging task for anti-tick vaccine developers. From the early 1980s, Cuba made enormous efforts to develop biotechnology [155,156]. A research group at the Cuban Center for Genetic Engineering and Biotechnology (CIGB), founded in 1986, began a project to develop new tools to approach tick control in the country.

After Willadsen discovered the protective capacity of the concealed antigen Bm86 from *R. microplus* ticks [157,158,159], a revolution in the development of vaccines against ectoparasites took place. Cattle immunization with the Bm86 protein showed reduced blood-meal volume, decreased tick engorgement, oviposition impairment and reduced egg viability like the effect of the naturally acquired host resistance against tick is manifested [160]. In the early 1990s, the Cuban biotechnology group at the CIGB obtained high expression levels of Bm86 antigen in recombinant *Pichia pastoris* yeast. The expression of Bm86 in yeast was associated with the formation of Bm86-containing particles that were highly immunogenic in cattle [161,162].

This Bm86 protein was used to develop the vaccine Gavac^TM^. This vaccine proved to be an effective control tool for acaricide-resistant or nonresistant tick strains under field conditions in Cuba [163], Brazil [164], Mexico [165], and Colombia [166]. The Gavac^TM^ vaccine was registered and commercialized in Cuba, Venezuela, Nicaragua, Panamá, México, Colombia, and Brazil. Currently, it is in the registration process in Costa Rica, Uruguay, Paraguay, and Bolivia. The initial vaccination schedule consisted of three immunizations on weeks 0, 4, and 7. Thereafter, re-immunizations every six months are needed to keep protective antibody titers against the Bm86 independently of the cattle breed, sex, age, or reproductive category [167]. Later studies demonstrated that two initial doses of Gavac^TM^ were sufficient to develop protective anti-Bm86 antibody titters and affect the reproductive performance of *R. microplus* females in field conditions, and the sanitary registers of Gavac^TM^ were accordingly modified [167].

After being registered in 1998, Gavac^TM^ was included in the CNPITC [168]. From the economical point of view, the overall effect obtained by Gavac^TM^ vaccine application is a significant reduction in the cost of the ticks and CTF disease control [169]. In addition, a study using dogs and *E. canis*/*B. canis* as models demonstrated that Gavac^TM^ vaccination diminishes TBDs in dogs, not only by a tick exposure reduction but also by decreasing the tick vector capacity, which extends the possibilities of using this vaccine [169]. Currently, tick eradication on pets is addressed using chemicals and acaricide products. However, resistance development of tick species parasitizing pets is also a big drawback and has been largely reported around the world [170,171,172,173]. As a future option to get the tick control on pets, vaccines and more integrated technologies could be required. 

The tick research team at the CIGB has carried out investigations with acidic ribosomal protein P0 as antigen target for an anti-tick vaccine [174,175,176]. Their results have shown P0 as a promising vaccine candidate that could be effective against different tick species. However, the identification and characterization of a protective antigen is not enough to deliver a commercially viable vaccine to the market. The production of this antigen in an immunologically effective way and the vaccine validation in a field situation are essential and expensive processes that must be addressed before this occurs. It should be noted that no other vaccine against ticks has reached the commercial stage after Bm86-based vaccines, largely reflecting the experimental difficulty of such investigations. Finding a carrier protein and a suitable adjuvant for releasing and presenting P0 antigen (pP0) to the immune system of a specific host is the current challenge of a P0-based vaccine. Very recently, pP0 was chemically conjugated to Bm86 as a carrier protein (carrying from 1 to 18 molecules of pP0 per molecule of Bm86) [177]. In that study, high immunogenicity and efficacy were achieved when dogs and cattle were vaccinated with the pP0–Bm86 conjugate and challenged with *R. sanguineus* s.l. and *R. microplus*, respectively. These promising results encourage the development of this antigen as a potential anti-tick vaccine. Dose and immunization schedule studies, optimization of the antigen’s production process, and the development of a robust analytical tool for the antigen quality control will be also necessary before proceeding with clinical assays.

## 13. Programs for the Control of Ticks and TBDs in Cattle in Cuba

Control of ticks and TBDs was one of the main tasks the Cuban Institute of Veterinary Medicine (IMV) since its creation in 1967. Subsequently, this activity was coordinated by the Experimental Station of Parasitology, created in 1982, which later became in the current National Laboratory of Parasitology (LNP) [17]. Initially, control of tick populations was based exclusively in the use of several classes of chemical acaricides, with high importation costs. However, resistance of tick populations to these chemicals was detected in the early ‘90s, a phenomenon that quickly arose a global emergency [178]. As an alternative, the integrated tick control strategy was adopted in Cuba, consisting of the systematic combination of at least two control technologies aiming to reduce selection pressure in favor of acaricide-resistant individuals, while maintaining adequate levels of animal production [179,180]. The Cuban National Program for Integrated Tick Control (CNPITC) was implemented all over the country in 1996.

The CNPITC was designed for integrated management of the available resources, combined harmonically to achieve economically-acceptable levels of tick infestation on the animals instead of tick eradication, while keeping endemic stability of CTF disease. Specifically, the CNPITC includes four types of technologies fundamentally: (I) selective breeding, (II) farming practices, (III) vaccines, and (IV) strategic use of acaricides. The main objectives set with the implementation of this program CNPITC were: (1) to reduce the intensity of tick infestation to less than ten engorged females per animal, (2) to reduce the use of chemicals and acaricides, (3) to lengthen the period between acaricide applications, (4) to prevent resistance to acaricides in tick populations, (5) to maintain constant and low inoculation rate of CTF pathogens, and (6) to maintain the endemic stability of the CTF disease, avoiding CTF outbreaks and clinical cases.

Since the 1960s, a nationwide program of genetic improvement (NPGI) for livestock was implemented in Cuba, using introgression of tick resistance and rusticity from *Bos indicus* breeds to low resistance but productive *B. taurus* breeds. The NPGI started with the F1 Holstein/Zebu, to develop autochthonous breeds as Holstein tropical (31/32 H × 1/32 Z), Siboney (5/8 H × 3/8 Z), Mambí (3/4 H × ¼ Z), or beef cattle breed, such as Chacuba (5/8 Charolais × 3/8 Z) and Crimousin (3/4 Limousin × 1/4 Criollo) [181]. The beginning of the CNPTIT coincided with the reorganization of the national strategy for genetic improvement of livestock, consisting of, among others, the extension of the Siboney herds across the country [90], based on its productivity and adaptability to the Cuban tropical environment [182]. In 2008, the Siboney had 58% preponderance on the nationwide livestock, and a tendency to increase [183].

Several transformations occurred in the cattle-raising system in Cuba in the early 1990s [90], which contributed significantly to the efficacy of the CNPITC. There was a change in the concentration of the cattle herds. In 1990, 80% of cattle were concentrated in 106 large specialized companies, distributed in dairy farms with 120 or 288 cows, while the beef cattle, in which 30% were in large feedlots of 5, 10, and 20,000 animals, and 20% were in the restricted grazing system in herds with 320 animals. Then, in 1993, the Basic Units of Cooperative Production (UBPC) and other small productive groups were created, in a strategy of decentralization and self-sustainability of the livestock sector [90]. The new UBPCs had an average extension of 1600 ha, with herds of 10–60 cows, and a density of 1.2 a 2.0 animal ha^−1^. This transformation contributed to reducing the population of ticks in the livestock areas; furthermore, this allowed a better organization of the pastures rotation to prevent accumulation of infective larvae and tick-host encounter. Besides, with this rearing system, artificial feeding of calves was eliminated, allowing early exposure to CTF pathogens for long-lasting immunity.

The vaccination with Gavac^TM^ constitutes a core element of the CNPITC, in which the anti-Bm86 acquired immunity in cattle is combined with the rational use of acaricides [168]. Ticks exposed to anti-Bm86 antibodies have reduced reproductive performance, which leads to a significant decrease in tick populations after two or three generations [167]. The vaccination schedule of Gavac^TM^ consists of two initial doses (2 mL each), inoculated by deep intramuscular injection, with an interval of four weeks, and then revaccination every six months of all animals under the immunization plan. Acaricide treatments are applied only when more than 10 engorged female ticks are found on the animals. This system reduces the frequency of acaricide use and the exposure of ticks to acaricides, which in turn reduces the emergence of acaricide-resistant tick strains, decreases the environmental pollution, and increases the useful lifetime of an acaricide.

After more than twenty years of the program’s application, the incidence of CTF diseases in Cuba has been reduced. However, the program is rigorously monitored nationwide, including annual planning of blood and tick samplings in all the municipalities across the country. This measure is particularly necessary because of the threatening expansion of *A. variegatum* tick and heartwater disease in the Caribbean region [124,184]. On the other hand, the vaccination scheduled requires technological discipline in its application, timely supply of the drug, and refrigerated transportation and storage. Currently, only around 50% of cattle are covered by the vaccination plan [88]; therefore, there is intention to extend it. In addition, the decentralization of livestock production has imposed a challenge for the NPGI in the country, creating a trend toward uncontrolled crossbreeding, with introgression of Holstein genes, which has been detected in several territories. This evidence supports the need for systematic reviews of the CNPITC in each municipality.

## Figures and Tables

**Table 1:** Tick species from family Argasidae reported in Cuba, host and geographical distribution.

Species	Main Hosts	Geographic Area (Provinces)
*Argas persicus*	Fowls	Artemisa, Mayabeque, Camagüey
*Argas miniatus*	Fowls	Pinar del Río, Camagüey
*Argas radiatus*	Fowls	Isla de la Juventud, Camagüey
*Antricola silvai*	Bat guano/Bats	Sancti Spiritus
*Antricola granasi*	Bat guano/Bats	Sancti Spiritus
*Antricola habanensis*	Bat guano/Bats	Mayabeque
*Antricola cernyi*	Bat guano/Bats	Cienfuegos
*Antricola occidentalis*	Bat guano/Bats	Pinar del Río
*Antricola martelorum*	Bat guano/Bats	Mayabeque
*Antricola naomiae*	Bat guano/Bats	Matanzas
*Antricola armasi*	Bat guano/Bats	Pinar del Río
*Antricola centralis*	Bat guano/Bats	Villa Clara
*Antricola siboneyi*	Bat guano/Bats	Santiago de Cuba
*Antricola marginatus*	Bat guano/Bats	Widespread in Cuba
*Ornithodoros azteci*	Bats	Mayabeque, Isla de la Juventud
*Ornithodoros brodyi*	Bats	Pinar del Río, Artemisa, Cienfuegos
*Ornithodoros cyclurae*	Reptiles	Granma
*Ornithodoros denmarki*	Birds	Matanzas
*Ornithodoros dusbabeki*	Bats	Isla de la Juventud
*Ornithodoros kelleyi*	Bats	Sancti Spiritus
*Ornithodoros natalinus*	Bats	Isla de la Juventud
*Ornithodoros capensis ^1^*	Birds	Pinar del Río
*Ornithodoros tadaridae*	Bats	Camagüey
*Ornithodoros viguerasi*	Bats	Pinar del Río, Artemisa, Mayabeque, Matanzas, Villa Clara, Sancti Spiritus, Santiago de Cuba, Guantánamo
*Otobius megnini*	Equine	Artemisa

^1^ These tick species are also considered as species complexes.

**Table 2:** Tick species from family *Ixodidae* reported in Cuba, host and geographical distribution.

Species	Main hosts	Geographic Area (Provinces)
*Amblyomma albopictum*	Reptiles, rodents	Artemisa, Mayabeque, Isla de La Juventud, Camagüey
*Amblyomma cajennense ^1^*	Domestic animals, humans	Pinar del Río, Artemisa, Mayabeque, Isla de la Juventud, Camagüey, Santiago de Cuba
*Amblyomma dissimile*	Reptiles, amphibians	Pinar del Río, Artemisa, Mayabeque, Isla de la Juventud, Santiago de Cuba
*Amblyomma quadricavum ^1^*	Reptiles, amphibians	Pinar del Río, Artemisa, Mayabeque, Cienfuegos
*Amblyomma torrei*	Reptiles, amphibians	Pinar del Río, La Habana, Camagüey
*Dermacentor nitens*	Equines	Widespread in Cuba
*Ixodes capromydis ^2^*	Cuban hutia	Isla de la Juventud
*Rhipicephalus microplus*	Bovines, equines	Widespread in Cuba
*Rhipicephalus sanguineus ^3^*	Domestic dogs	Widespread in Cuba

^1^ These tick species are also considered as species complexes. For example, the *Amblyomma cajennense* species complex (or *A. cajennense* sensu lato) is formed by six species, namely *A. cajennense sensu stricto*, *Amblyomma sculptum*, *Amblyomma mixtum Amblyomma tonellia*, *Amblyomma patinoi*, and *Amblyomma interandinum* [49,50]. From these species, only *A. mixtum* has been reported in Cuba [50,51]. ^2^ Species endemics to Cuba. ^3^ The preferred host of this tick is the domestic dog. However, in the absence of dogs, it can feed on humans.

**Table 3:** TBPs detected in Cuba and methods used in their diagnosis.

Pathogens	Reported Host	Detection Method	References
*A. marginale, B. bovis, B. bigemina*	cattle	CFT, CAT	Rodríguez et al., 1980 [138]
*A. marginale, B. bovis, B. bigemina*	cattle	Morphological diagnosis	Salabarría and Jiménez, 1983 [133]
*M. ovis*	sheep, goat	Morphological diagnosis	Joa et al., 1987 [126]
*A. ovis, B. ovis*, *M. ovis*, *B. motasi*	sheep, goat	Morphological diagnosis	Rodriguez et al., 1989 [129]
*Babesia* spp.	cattle	IFA	Alonso et al., 1988 [136]
*B. bovis, B. bigemina*	cattle	IPT	Blandino et al., 1992 [137]
*A. marginale, B. bovis, B. bigemina*	cattle	CFT, CAT, IFA	Fadraga et al., 1991 [86]
*B. bovis*	cattle	ELISA	Blandino et al., 1998 [136]
*A. marginale*	cattle	PCR	Corona et al., 2011 [139]
*B. bovis*	buffalo	nPCR	Obregón et al., 2012 [99]
*A. marginale*	buffalo	nPCR	Corona et al., 2012 [140]
*B. burgdorferi* s.l. *Anaplasma-Ehrlichia*, *Babesia-Theileria*	*D. nitens*, *A. cajennense* s.l., *R. microplus*	Multiplex PCR	Rodríguez et al., 2015 [26]
*B. bovis, B. bigemina*	buffalo	SYBR Green qPCR	Obregón et al., 2016 [101]
*R. amblyommii*	*A. mixtum*	PCR	Noda et al., 2016 [79]
*E. canis, B. canis vogeli*	*R. sanguineus* s.l.	PCR, nPCR	Gonzalez et al., 2016 [118]
*C. burnetii*	*A. mixtum*	PCR	Noda et al., 2016 [51]
*A. platys*	*R. sanguineus* s.l., dog	nPCR	Silva et al., 2016 [116]
*B. caballi, T. equi*	horse	nPCR	Díaz-Sánchez et al., 2018 [24]
*E. canis*	dog	iELISA, nPCR	Gonzalez et al., 2018 [25]
*A. marginale*	buffalo	TaqMan qPCR	Obregón et al., 2018 [100]
*A. marginale, T. annulata*	cattle, buffalo	Multiplex TaqMan qPCR	Díaz-Sánchez et al., 2019 [14]
*B. bovis, B. bigemina, A. marginale*	buffalo	iELISA, nPCR	Obregón et al., 2019 [21]
*M. wenyonii,* Candidatus *M. haemobus*	cattle, buffalo	TaqMan qPCR	Díaz-Sánchez et al., 2019 [141]
*B. bovis, B. bigemina*	cattle, buffalo, *R. microplus*	SYBR Green qPCR	Obregón et al., 2020 [70]
